# Identification of a low-risk subgroup of HER-2-positive breast cancer by the 70-gene prognosis signature

**DOI:** 10.1038/sj.bjc.6605916

**Published:** 2010-11-16

**Authors:** M Knauer, F Cardoso, J Wesseling, P L Bedard, S C Linn, E J T Rutgers, L J van 't Veer

**Affiliations:** 1 Division of Diagnostic Oncology, The Netherlands Cancer Institute/Antoni van Leeuwenhoek Hospital, Plesmanlaan 121, Amsterdam NL-1066CX, The Netherlands; 2 Department of General and Thoracic Surgery, Academic Teaching Hospital, Feldkirch, Austria; 3 Department of Medicine, Institut Jules Bordet, 121 Boulevard de Waterloolaan, Brussels B-1000, Belgium; 4 TRANSBIG Consortium, Brussels, Belgium

**Keywords:** breast cancer, gene expression profiling, MammaPrint, risk assessment, HER-2, adjuvant chemotherapy

## Abstract

**Background:**

Overexpression of HER-2 is observed in 15–25% of breast cancers, and is associated with increased risk of recurrence. Current guidelines recommend trastuzumab and chemotherapy for most HER-2-positive patients. However, the majority of patients does not recur and might thus be overtreated with adjuvant systemic therapy. We investigated whether the 70-gene MammaPrint signature identifies HER-2-positive patients with favourable outcome.

**Methods:**

In all, 168 T1–3, N0–1, HER-2-positive patients were identified from a pooled database, classified by the 70-gene signature as good or poor prognosis, and correlated with long-term outcome. A total of 89 of these patients did not receive adjuvant chemotherapy.

**Results:**

In the group of 89 chemotherapy-naive patients, after a median follow-up of 7.4 years, 35 (39%) distant recurrences and 29 (33%) breast cancer-specific deaths occurred. The 70-gene signature classified 20 (22%) patients as good prognosis, with 10-year distant disease-free survival (DDFS) of 84%, compared with 69 (78%) poor prognosis patients with 10-year DDFS of 55%. The estimated hazard ratios (HRs) were 4.5 (95% confidence interval (CI) 1.1–18.7, *P*=0.04) and 3.8 (95% CI 0.9–15.8, *P*=0.07) for DDFS and breast cancer-specific survival (BCSS), respectively. In multivariate analysis adjusted for known prognostic factors and hormonal therapy, HRs were 5.8 (95% CI 1.3–26.7, *P*=0.03) and 4.7 (95% CI 1.0–21.7, *P*=0.05) for DDFS and BCSS, respectively.

**Interpretation:**

The 70-gene prognosis signature is an independent prognostic indicator that identifies a subgroup of HER-2-positive early breast cancer with a favourable long-term outcome.

Overexpression of HER-2 is observed in 15–25% of invasive breast cancers, and it is considered a negative prognostic factor. Most HER-2-positive breast cancer patients are deemed to be at a high risk of relapse by current treatment guidelines and thus, nearly all patients are allocated to (neo)adjuvant trastuzumab and chemotherapy. According to the National Comprehensive Cancer Network (2009) practise guidelines in oncology, chemotherapy and trastuzumab are advised for all HER-2-positive lymph node-positive tumours and all HER-2-positive node-negative tumours larger than 1 cm. Even for moderately or poorly differentiated tumours 0.6–1 cm, this combined treatment should be considered. For hormone receptor-negative HER-2-positive tumours, the indication for chemotherapy with or without trastuzumab is expanded further to include tumours smaller than 0.5 cm, if lymph node micrometastases are present. Endocrine therapy should also be given in patients with hormonal receptor-positive disease. The St. Gallen International consensus conference 2007 considered all patients with HER-2 overexpression at least as intermediate risk, regardless of tumour size, nodal status or other clinical risk factors. Nevertheless, it was stated that trastuzumab ‘should not be viewed as standard treatment in women with a primary tumour <1 cm of size and with no axillary involvement, particularly in patients with highly and perhaps also incompletely endocrine responsive disease’ (Goldhirsch *et al*, 2007, 2009).

Trastuzumab is expensive, requires prolonged course of treatment and carries a small but potentially serious risk of cardiac toxicity that can result in permanent disability. The aggregate data from the adjuvant trastuzumab trials indicate that the majority of patients with HER-2-positive disease remain disease-free even in the absence of trastuzumab therapy. This proportion was 74% (1377 out of 1698) at 3 years follow-up in the HERA trial (HERceptin adjuvant; Untch *et al*, 2008). In addition, analysis of molecular subtypes of breast cancer demonstrated a ∼40% relapse-free survival rate in node-negative, HER-2-positive patients who had not received any systemic therapy at all, with a median follow-up time of 9 years (Parker *et al*, 2009). The identification of a good prognosis subgroup of HER-2-positive patients, for whom chemotherapy and/or trastuzumab could safely be omitted, might reduce both unnecessary toxicity and high treatment costs. In this study, the 70-gene prognosis signature (MammaPrint, Agendia Inc., Huntington Beach, CA, USA and Amsterdam, The Netherlands), which is validated as an independent prognostic indicator for node-negative and 1–3 node-positive disease, regardless of oestrogen receptor (ER) status (van ’t Veer *et al*, 2002; van de Vijver *et al*, 2002; Buyse *et al*, 2006; Bueno-de-Mesquita *et al*, 2008; Mook *et al*, 2008), was used to explore whether a subgroup of HER-2-positive breast cancer patients with a favourable long-term outcome could be distinguished.

## Materials and methods

### Patient population

A database with individual patient data for 1288 patients with known HER-2 status, 70-gene prognosis profile testing results, and long-term outcome was pooled from previous studies (van de Vijver *et al*, 2002; Bueno-de-Mesquita *et al*, 2007, 2008; Kok *et al*, 2008; Mook *et al*, 2008). Tumour grading was performed according to the Bloom–Richardson method. The ER and PR status were determined by immunohistochemistry (IHC) and interpreted as positive if 10% or more of the cells were stained. Histological grading and hormone receptor status were centrally determined for each involved study by experienced breast pathologists.

The study patients were selected from the pooled database according to the following criteria: unilateral stage pT1–3, N0–1, M0, HER-2-positive invasive breast carcinoma diagnosed between 1984 and 2006, no neoadjuvant chemotherapy and surgical treatment with either breast-conserving therapy or mastectomy with sentinel node biopsy or axillary lymph node dissection. This was followed by radiotherapy and adjuvant systemic therapy if indicated by local treatment guidelines, taking into account patient preference and consent. Detailed inclusion criteria for each of the previous studies have been previously described. All of the studies were approved by the responsible institutional review boards (van de Vijver *et al*, 2002; Bueno-de-Mesquita *et al*, 2007, 2008; Kok *et al*, 2008; Mook *et al*, 2008).

### Assessment of HER-2

The overexpression of HER-2 was defined as either a 3+ staining score by IHC or by 2+ score and a positive chromogenic *in situ* hybridisation (CISH) assay for gene amplification. The majority of HER-2-positive cases had been determined either at the European Institute of Oncology, Milan (9 cases) or at the pathology department of the Netherlands Cancer Institute (NKI), Amsterdam (121 cases). However, the rate of discordance between local and central pathology was 32% (12 out of 38 cases from different local pathology laboratories). Overall we found a 10% discordance rate (17 out of 168) between original and confirmation assessment of HER-2 status. Recently, a very high concordance of 95% regarding HER-2 assessment between the NKI laboratory and an independent College of American Pathologists (CAP)-accredited US reference laboratory using Food and Drug Administration-approved procedures and American Society of Clinical Oncology/CAP guidelines has been demonstrated (Roepman *et al*, 2009).

### Molecular analyses

RNA of the frozen tumour samples was extracted, analysed and classified by the 70-gene MammaPrint signature as either good or poor prognosis at the FDA-cleared and CLIA-accredited Agendia Laboratories as previously described (van ’t Veer *et al*, 2002; Glas *et al*, 2006). In all, 5 out of 168 tumour samples (3%) used for this analysis had been used for the development of the 70-gene signature.

### Statistical analyses

Time-to-event analyses using updated and centrally verified individual patient data were performed using the pooled database (Microsoft Access; Microsoft, Redmond, WA, USA). End points considered were distant disease-free survival (DDFS), defined as time from surgery to any distant metastasis and breast cancer specific survival (BCSS), defined as time from surgery to breast cancer-related death. Kaplan–Meier survival plots and log-rank tests were used to assess differences in DDFS and BCSS of the 70-gene profile good and poor prognosis groups. All *P*-values are two-sided and were considered statistically significant if less than 0.05. The adjusted hazard ratios (HRs) and the corresponding 95% confidence intervals (95% CI) for all reported analyses were derived from Cox proportional hazards models. Covariates used in adjusted models included age at diagnosis, tumour size, number of positive lymph nodes, histological grade, ER and PR status, and hormonal therapy. All statistical analyses were performed with SPSS 15.0 for Windows (SPSS Inc., Chicago, IL, USA).

### Role of the funding source

The Austrian Society of Surgery and Agendia BV provided unrestricted educational grants for the work of M Knauer, and were not involved in study design, collection, analysis or interpretation of data, writing neither of the report nor in the decision to submit the manuscript.

## Results

**Figure 1:**
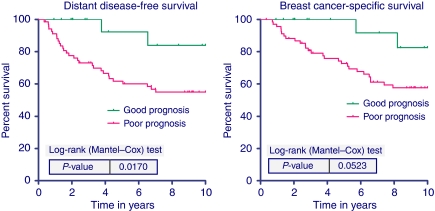
Ten-year distant disease-free survival (left) and breast cancer-specific survival (right) according to the 70-gene signature for the 89 HER-2-positive patients who did not receive chemotherapy or trastuzumab.

Detailed tumour and treatment characteristics of the HER-2-positive study population are shown in Table 1. After a median follow-up of 65 months (range 4–303), 49 (29%) distant recurrences and 41 (24%) breast cancer-specific deaths occurred in the 168 HER-2-positive patients. We focused our analyses on patients who did not receive adjuvant chemotherapy or trastuzumab to assess the prognostic value of the 70-gene signature in a more homogeneous group of HER-2-positive patients. In this group of 89 patients without adjuvant chemotherapy, 36 patients (40%) received adjuvant endocrine treatment. The 70-gene prognosis signature classified 20 (22%) patients as good prognosis, with 10-year distant DDFS of 84%, compared with 69 (78%) poor prognosis patients with 10-year DDFS of 55%. The estimated HRs were 4.5 (95% CI 1.1–18.7, *P*=0.04) and 3.8 (95% CI 0.9–15.8, *P*=0.07) for DDFS and BCSS, respectively. The corresponding Kaplan–Meier curves with log rank *P*-values for DDFS and BCSS are shown in Figure 1.

The HER-2-positive cases characterised as 70-gene good prognosis were more likely to have lobular cancers (26 *vs* 4%, *P*=0.002), and were less likely to be histological grade 3 (21 *vs* 71%, *P*<0.001). No other statistically significant differences were found between the groups regarding the other baseline characteristics, including age, T-stage, lymph node involvement, ER and PR status, and type of surgery. Significantly fewer patients received adjuvant endocrine therapy in the good prognosis group compared with the poor prognosis group: 16 out of 20 patients (80%) received no endocrine treatment, whereas in the poor prognosis group 37 out of 69 (54%) did not receive endocrine therapy (*P*=0.041).

In multivariate analysis, adjusted for known prognostic factors and endocrine treatment as described above, only the 70-gene signature and tumour size were independently correlated with 10-year distant metastasis-free and BCSS. Detailed results of the multivariate analyses are shown in Table 2.

**Figure 2:**
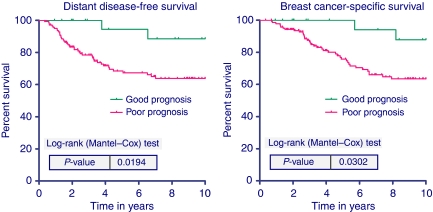
Ten-year distant disease-free survival (left) and breast cancer-specific survival (right) according to the 70-gene signature for all 168 HER-2-positive breast cancer patients.

In the more heterogeneously treated group of all 168 patients, including 79 patients treated with adjuvant chemotherapy or trastuzumab, the univariate HR for good *vs* poor prognosis by the 70-gene signature for 10-year DDFS was 4.6 (1.1–19.2, *P*=0.034). For BCSS, the HR was 4.2 (1.0–17.5, *P*=0.047). The Kaplan–Meier survival curves for this group are shown in Figure 2.

**Figure 3:**
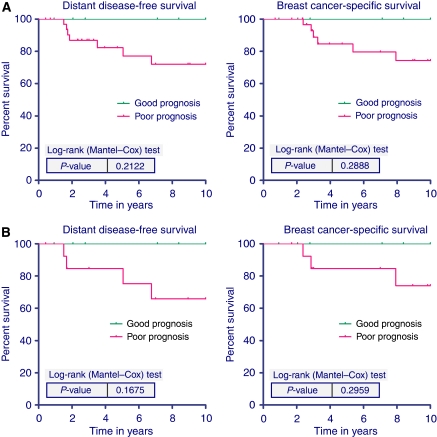
(**A**) Ten-year distant disease-free survival (left) and breast cancer-specific survival (right) according to the 70-gene signature for the 40 patients with HER-2 positive and highly endocrine-responsive tumours according to the St. Gallen criteria (>50% ER positive and >50% PR positive). Out of the 11 low-risk patients, 7 were untreated, 4 received chemotherapy and one of these patients received trastuzumab. (**B**) Ten-year DDFS (left) and BCSS (right) for the 21 chemotherapy-naive patients.

An exploratory subgroup analysis was performed according to the degree of hormonal receptor expression in this group of patients with HER-2-positive disease. To explore whether expression of hormonal receptors identified a subgroup of HER-2-positive/70-gene profile low-risk tumours with a particularly favourable outcome, we separately analysed the 40 out of 168 patients (24%) with HER-2 overexpression that were highly endocrine responsive (see Kaplan–Meier curves in Figure 3A). In all, 21 out of these 40 patients did not receive adjuvant chemotherapy, shown in Figure 3B. According to the 2007 St. Gallen consensus conference, ‘highly endocrine responsive’ tumours express high levels of both steroid hormone receptors in a majority of cells (Goldhirsch *et al*, 2007). For this analysis, IHC staining of both ER and PR in 50% or more of cells were considered ‘highly endocrine-responsive’. Within this subgroup, no distant metastases or breast cancer-specific deaths were observed in the patients with a 70-gene profile ‘good prognosis’ testing result (11 out of 40; 28%). Figure 3A depicts the survival curves for the 40 highly endocrine-responsive patients and Figure 3B shows 10-year DDFS and BCSS for the 21 chemotherapy-naive patients within this subgroup.

## Discussion

This is the first study to demonstrate the existence of a clinically significant subgroup of patients with HER-2-positive early breast cancer, who experience a favourable long-term outcome that can be identified with a validated and commercially available gene expression assay. The 70-gene MammaPrint signature was a strong independent prognostic indicator for patients with HER-2-positive disease in the absence of adjuvant chemotherapy and/or trastuzumab. Furthermore, in low-risk and highly endocrine-responsive tumours, no relapses or cancer-related deaths were observed in the patients classified as good prognosis by the genomic assay.

One of the clear strengths of this study is the methodological approach of a pooled analysis of updated, individual patient data, which allows for appropriate time-to-event analyses by combining data from different series into one study (Clarke and Stewart, 1997). Furthermore, HER-2 status of all patients in the respective series had been centrally determined or reviewed by experienced breast cancer pathologists during the study periods. To be deemed HER-2-positive for this study, tumours demonstrated either 3+ IHC staining or 2+ IHC staining and a positive CISH result. In the good prognosis group, HER-2 overexpression was confirmed for all patients by CISH, regardless of the IHC result. However, the small number of patients, the limited follow-up time for some patients and the somewhat heterogeneous group of patients with or without endocrine therapy constitute the major limitations of this analysis. The significant differences between the groups regarding histological grade reflect the differences in the function of genes represented in the 70-gene signature, that is to say the proliferation cluster. Furthermore multivariate analyses of retrospective data cannot account for all potential biases in treatment selection and these data require confirmation in other datasets and clinical studies. For instance, this subgroup is currently being evaluated in the ongoing MINDACT trial. The trial will prospectively evaluate whether it is acceptable to withhold chemotherapy in these low-risk patients, and subsequent studies will be undertaken by the TRANSBIG group and others to further characterise the differences between the genomic high-risk and low-risk groups. This will include whole-genome arrays of these patients, analysis of the phosphoinositide-3-kinase pathway and additional pathways, and grading of histological specimens for the degree of lymphocyte infiltration by unbiased breast pathologists.

Alexe *et al* (2007) used an existing microarray dataset of 286 early breast cancers by Wang *et al* (2005), available from the National Center for Biotechnology Information GenBank GEO database. The authors characterised two HER-2-positive subtypes based on the *in silico* analysis of 42 tumour samples with increased expression of HER-2-related genes, with the low recurrence subtype being associated with a relative overexpression of lymphocyte-associated genes and prominent lymphocytic infiltration on histological analysis of 13 independent tumours, of which only 10 were available for evaluation. This hypothesis-generating study by Alexe *et al* relied on the identification of HER-2-positive genes by the expression level of genes in the 17q12 amplicon, and the authors did not confirm HER-2 positivity with traditional measures of HER-2 assessment. The authors stated that stratification by the 70-gene signature was not able to discriminate high- and low-risk HER-2 subtypes and therefore, may not provide any additional prognostic information within the HER-2-positive subgroup. In comparison, in this analysis of 1288 patients of which 168 were HER-2 positive, we found two clearly different subtypes of HER-2-positive breast cancer. These subtypes with good or poor prognosis were distinguished by a validated assay, which is available in daily practise. These results from pooled individual patient data and the original 70-gene readouts are in our opinion more robust compared with both the analysis in the Alexe paper as well as the recently published *in silico* meta-analysis by Desmedt *et al* (2008). In their study, suboptimal data normalisation and readout from a different microarray platform might have contributed to the finding that all prognostic signatures failed to discriminate good *vs* poor outcome in the HER-2-positive cluster. A similar analysis was performed by Wirapati *et al* (2008) with several publicly available gene expression datasets. In both papers HER-2 positivity was determined by molecular classification of HER-2-associated genes rather than traditional measures of HER-2 protein overexpression and/or gene amplification.

Before the advent of expression profiling, Harbeck *et al* (1999) and Zemzoum *et al* (2003) investigated urokinase-type plasminogen activator (uPA), and its inhibitor PAI-1 as prognostic factor for HER-2-positive breast cancer. The authors showed that uPA/PAI-1 levels in primary tumour tissue indicated an aggressive course of disease in lymph node-negative breast cancer independent of HER-2 status. The relationship between tumours identified as low risk by uPA/PA-1 and the 70-gene profile is unknown.

The second widely used prognostic tool is the 21-gene recurrence score (Oncotype DX, Genomic Health Inc., Redwood City, CA, USA; Paik *et al*, 2004), which is based on real-time RT–PCR and uses formalin-fixed, paraffin-embedded tissue, and is retrospectively validated for ER-positive breast cancer. As the measurement of the expression of the HER-2 gene itself was chosen as important contributing factor in this ‘knowledge-driven approach’, most if not all HER-2-positive tumours are classified as intermediate or high risk and therefore, this assay is unlikely to add prognostic information for HER-2-positive disease. Of the 55 HER-2-positive cases identified in the NSABP B-14 trial, 50 had a high recurrence score (RS) and 5 had an intermediate RS, respectively, whereas none of the patients was assigned to a low recurrence score (Paik *et al*, 2004; S Paik, personal communication). In comparison, the 70-gene signature was developed using the ‘data-driven approach’ with unbiased, genome-wide gene expression. The HER-2 gene itself was not on the list of the 70 priority genes selected solely on the basis of differences in gene expression levels from intact RNA of frozen tumours. This study suggests, that a clinically meaningful and larger proportion (22%) of chemotherapy-untreated HER-2-positive tumours are identified as low risk by the 70-gene profile, and these patients experience a favourable long-term outcome. This is especially remarkable, as 13 of 16 ER-positive low-risk patients did not receive endocrine treatment at the time the original studies have been conducted.

Avoiding overtreatment with chemotherapy and/or trastuzumab for truly low-risk HER-2-positive patients is an important goal, taking into account the risk of serious adverse events and the cost of these treatment regimens. Currently, trastuzumab monotherapy in the absence of chemotherapy is not regarded as standard of care for patients with HER-2-positive disease, although many experts at the St. Gallen consensus conference believed that trastuzumab alone may be reasonable for a subset of patients with HER-2-positive disease in the future (Goldhirsch *et al*, 2007). Our data raises the intriguing hypothesis that this strategy of anti-HER-2 therapy in combination with endocrine therapy might first be tested in patients with highly endocrine-responsive HER-2-positive disease and/or patients with hormonal receptor expression with a ‘good prognosis’ 70-gene profile. Recently, Chia *et al* (2008) reported similar findings, as the HER-2-positive, ER-positive subgroup of T1 cancers had a more favourable outcome with a 10-year BCSS of 92%, as compared with the HER-2-positive, ER-negative subgroup with a 10-year BCSS of only 76%.

In summary, our study suggests the existence of a low-risk HER-2-positive subgroup of patients with favourable outcome, which can be identified by the 70-gene MammaPrint gene signature. The results of this study support the evaluation of less intensive treatment strategies in this low-risk group. Further validation of this important finding is ongoing in the MINDACT trial, whereby patients with HER-2-positive disease deemed to be at a high clinical risk by Adjuvant!Online (Ravdin *et al*, 2001) with a ‘good prognosis’ MammaPrint profile may be randomised to receive no chemotherapy but may be treated with trastuzumab alone at the discretion of the treating physician.

## Figures and Tables

**Table 1:** Tumour characteristics and adjuvant treatment of patients with HER-2-positive breast cancer

**Characteristic**	**Patients without chemotherapy or trastuzumab (*n*=89)**	**All HER-2-positive patients (*n*=168)**
*Age*		
Median (range)	50 (28–79)	49 (28–79)
		
*Tumour size*		
T1	46 (52%)	89 (53%)
T2/3	43 (48%)	79 (47%)
		
*Lymph node status*		
N0	55 (62%)	96 (57%)
N1	34 (38%)	73 (43%)
		
*Histological grade*		
Grade 1/2	35 (39%)	64 (38%)
Grade 3	53 (60%)	104 (62%)
		
*ER status*		
Positive	57 (64%)	103 (61%)
Negative	32 (36%)	64 (38%)
		
*PR status*		
Positive	36 (40%)	70 (42%)
Negative	53 (60%)	98 (58%)
		
*Adjuvant treatment*		
Untreated	53 (60%)	53 (32%)
Hormonal therapy	36 (40%)	66 (39%)
Chemotherapy	0	77 (46%)
Trastuzumab	0	25 (15%)

Abbreviations: ER=oestrogen receptor; *n*=number of patients; PR=progesterone receptor.

**Table 2:** Results of the multivariate analysis by Cox proportional hazards models for 89 HER-2-positive patients without chemotherapy or trastuzumab

**Variable**	**HR**	**95% CI**	***P*-value**
*DDFS*			
*MammaPrint*	5.78	1.25–26.66	0.025
Age	0.99	0.95–1.04	0.697
*Tumour size*	1.07	1.03–1.12	0.001
Number of positive lymph nodes	1.09	0.70–1.70	0.694
Grade 1/2 *vs* 3	1.12	0.48–2.65	0.793
ER status	1.41	0.54–3.69	0.483
PR status	0.66	0.26–1.71	0.395
Adjuvant hormonal therapy	0.50	0.14–1.74	0.274
			
*BCSS*			
*MammaPrint*	4.70	1.01–21.75	0.048
Age	1.00	0.96–1.05	0.884
*Tumour size*	1.06	1.02–1.11	0.007
Number of positive lymph nodes	1.09	0.68–1.73	0.727
Grade 1/2 *vs* 3	1.28	0.52–3.20	0.592
ER status	1.27	0.47–3.41	0.639
PR status	0.66	0.24–1.78	0.409
Adjuvant hormonal therapy	0.36	0.09–1.37	0.133

Abbreviations: BCSS=breast cancer-specific survival at 10 years; CI=confidence interval; DDFS=distant disease-free survival at 10 years; ER=oestrogen receptor; HR=hazard ratio; PR=progesterone receptor.
